# Construction Notes

**Published:** 1896-09-05

**Authors:** 


### CONSTRUCTION NOTES.

### New Hospital—Aberdeen Royal Asylum.

The new hospital at the Royal Asylum is now open. The whole scheme reflects the highest credit on the architects, Messrs. Smith and Kelly, the carrying out of whose plans involved an expenditure of £24,000. The hospital, which is situated immediately behind the present building, is in the form of five distinct blocks, connected by a corridor, which extends from end to end of the buildings, and is 526 feet long. In the central position is the administrative block, which is three storeys in height. It contains, on the ground floor, a board-room and offices, porters' rooms, assistant medical officer's room, surgery, research room, two visitors' rooms, two admission rooms, one for male and one for female patients, with bathroom and clothes store attached to each; a spacious dining hall with open-timbered roof for the patients, with servery, and two sculleries adjoining; and two dining rooms for male and female nurses. The first floor of the block, reached by a staircase on each side, is divided in the middle, so as to completely separate the two halves. On the male side is the assistant medical officer's bedroom, a bath-room, two store-rooms, and seven other rooms to be used as day rooms and bedrooms for private patients. On the female side are the head nurse's rooms and other rooms similar to those on the male side. The second floor is divided in the centre, and contains bed-rooms, &c., for the night nurses. The dining hall on the ground floor is in the rear of the block. Occupying a central position, and with the main corridor at each end opening into it by a door, it is accessible from all parts of the hospital. On each side of the administrative block is a mental observation block for recent hopeful cases. These blocks, for males and females respectively, are two storeys in height, and contain on the ground floor, two day rooms. The larger room is intended for cases of morbid depression, and is designed to accommodate 21 patients. The smaller room is for the reception of cases of morbid excitement, and it will be capable of receiving 12 patients. The first floor of each observation block contains a dormitory calculated to accommodate 19 patients. Attached to the dormitory are three single rooms for special cases, and two rooms for nurses. The observation blocks are all connected with the administrative block by the main corridor in the rear, and by an exercise corridor in front. The space between the two corridors is occupied by a conservatory, where the patients can sit. About 40 feet beyond the observation blocks on each side stands a hospital block, extending at right angles to the general axis, with an extreme length of 189 feet. The hospital blocks are one storey high. Each of these blocks has, at its end, two sick wards and a day-room combined, for the reception of 22 patients. The main longtitudinal corridor divides the hospital blocks into two parts. The southern contains, between the sick wards and the main corridor, six single rooms, for cases requiring special treatment, several store-rooms, an attendants' room, opening into the sick ward, and a small kitchen. The northern portion of each hospital block consists of thirteen single rooms and a bath-room, arranged along the sides of the passage. The main corridor passing through each block is continued 25 feet beyond it, where it communicates with a small block for the isolation of infectious disease. An important feature in the new building is the system of heating and ventilation, which is on a plan supplied by the Steutevant Engineering Company, London. The buildings are lighted throughout by electric light. The buildings for the engineering purposes also comprise kitchens and laundry.

